# Chilaiditi Sign and Syndrome as a Finding in the Emergency Room: A Case Report

**DOI:** 10.7759/cureus.75674

**Published:** 2024-12-13

**Authors:** Elisabete Mendes, Rita Diz, Djenabu Cassama, Ana Dourado

**Affiliations:** 1 Internal Medicine Department, Unidade Local de Saúde do Alto Alentejano, Hospital Dr. José Maria Grande, Portalegre, PRT; 2 Internal Medicine Department, Unidade Local de Saúde do Nordeste, Unidade Hospitalar de Bragança, Bragança, PRT; 3 Clinical Pathology Department, Unidade Local de Saúde do Nordeste, Unidade Hospitalar de Bragança, Bragança, PRT

**Keywords:** chilaiditi sign, chilaiditi syndrome, constipation, differential diagnosis, radiography

## Abstract

The Chilaiditi sign is the presence of a loop of bowel interposed between the liver and the diaphragm. In most cases, the Chilaiditi sign is diagnosed as a rare incidental radiological finding on chest X-rays or the abdomen of asymptomatic patients. When associated with symptoms, it is named Chilaiditi syndrome. However, the symptoms are nonspecific, encompassing some of the most common causes of resorting to the emergency room (ER), such as abdominal pain, dyspnea, vomiting, or constipation, which hinders the differential diagnosis, so it's necessary to resort to imaging tests. We report a case of a 56-year-old male who presented to the ER due to dyspnea, constipation, and generalized abdominal pain/discomfort, with no other complaints. The chest X-ray revealed the presence of the Chilaiditi sign, and the diagnosis of Chilaiditi syndrome was considered as a diagnostic hypothesis and later confirmed with the computerized tomography (CT) scan and correlation of the patient's symptoms. We conclude that although Chilaiditi's syndrome is a rare condition, it should be considered as a possible differential diagnosis so that the patient receives adequate treatment with minimal harm since most cases are resolved with conservative treatment.

## Introduction

Prior to writing this article, the clinical case reported was presented on a poster at the European Congress of Internal Medicine held in June 2022. In this context, the abstract was also published in the European Journal of Case Reports in Internal Medicine in June 2022 [1].

The Chilaiditi sign was first described in 1910 by Greek radiologist Demetrius Chilaiditi [2] and refers to the temporary or permanent presence of a hollow viscus, such as the colon or small intestine, in the hepatodiaphragmatic space. It is typically an incidental and rare finding, but when associated with symptoms such as dyspepsia, nausea, vomiting, abdominal pain, respiratory symptoms, abdominal distension, constipation, intestinal obstruction, or subocclusion, it is referred to as Chilaiditi syndrome.

The exact cause of Chilaiditi syndrome is often unknown and likely multifactorial. Various factors can alter the anatomical relationship between the liver, colon, and diaphragm, facilitating the development of Chilaiditi syndrome. These predisposing factors can be categorized as hepatic factors (relaxation of liver ligaments, cirrhosis, hepatic atrophy, ascites), intestinal factors (megacolon, meteorism, abnormal colonic motility), and diaphragmatic factors (diaphragmatic thinning, phrenic nerve injury, changes in intrathoracic pressure such as in emphysema). Other factors described include chronic constipation, previous abdominal surgery, obesity, and aerophagia [3-5]. The anatomical relationships can be confirmed through an abdominal CT scan.

The treatment of Chilaiditi syndrome is usually conservative and includes weight loss, control of aerophagia and ascites, and changes in body position. However, in rare cases, surgical intervention with resection or, more commonly, fixation of the interposed viscus may be necessary. Cases of volvulus typically require emergency surgery with colectomy (in cases of perforation and gangrene) or colopexy. The laparoscopic approach has been preferred by surgeons due to less postoperative pain, fewer complications related to the surgical incision, a shorter hospital stay, and an earlier return to activities [5,6,7,8].

## Case presentation

We present the case of a 56-year-old male patient, independent in daily activities, with a history of cognitive deficit, asthma, hypertension, and obesity. He recurred to the emergency room with complaints of dyspnea, generalized abdominal discomfort, and constipation, without fever or other associated complaints.

**Figure 1:**
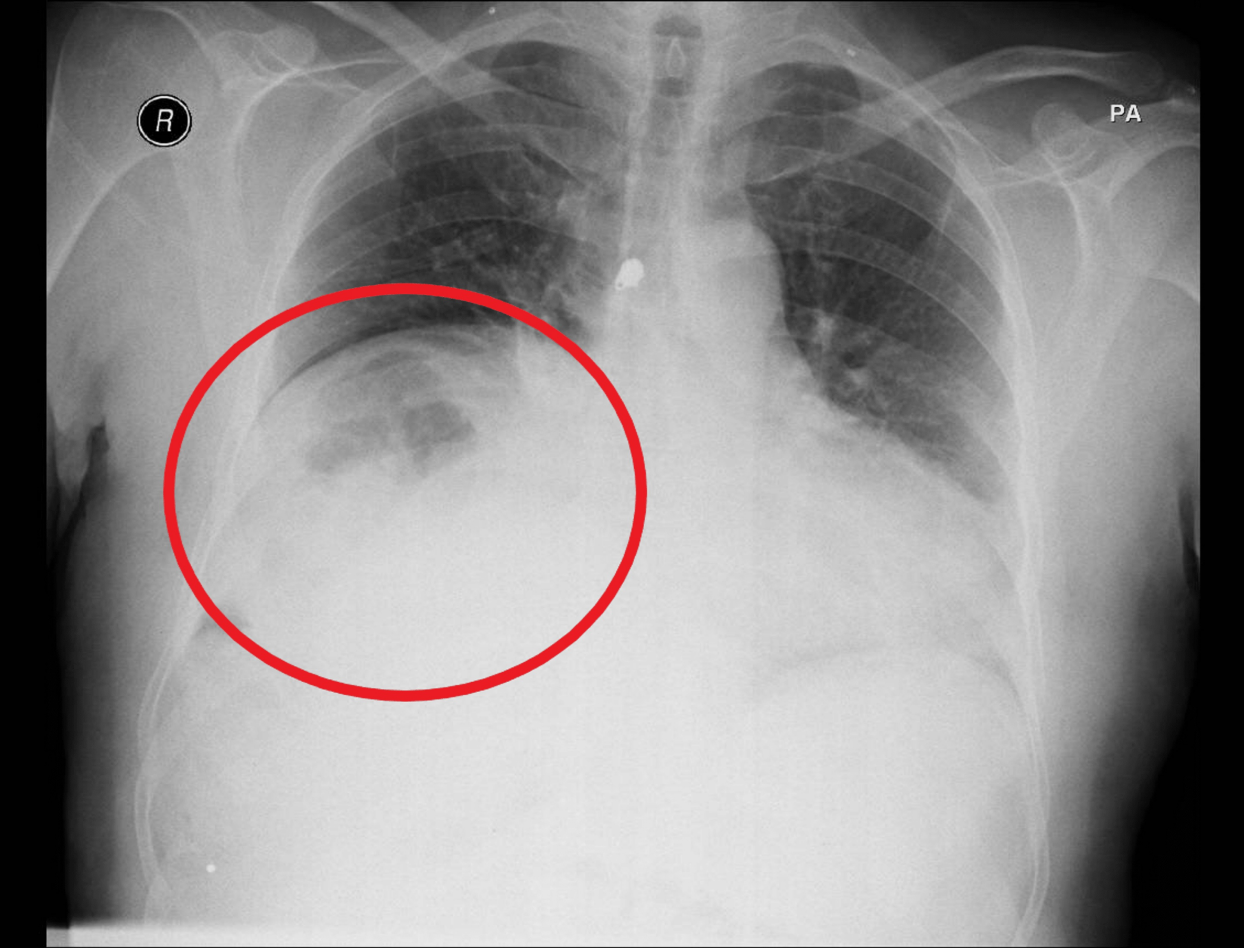
Chest X-ray The Chilaiditi sign is observed, characterized by the elevation of the right hemidiaphragm and interposition of the intestinal loop between the liver and the diaphragm (red circle).

**Figure 2:**
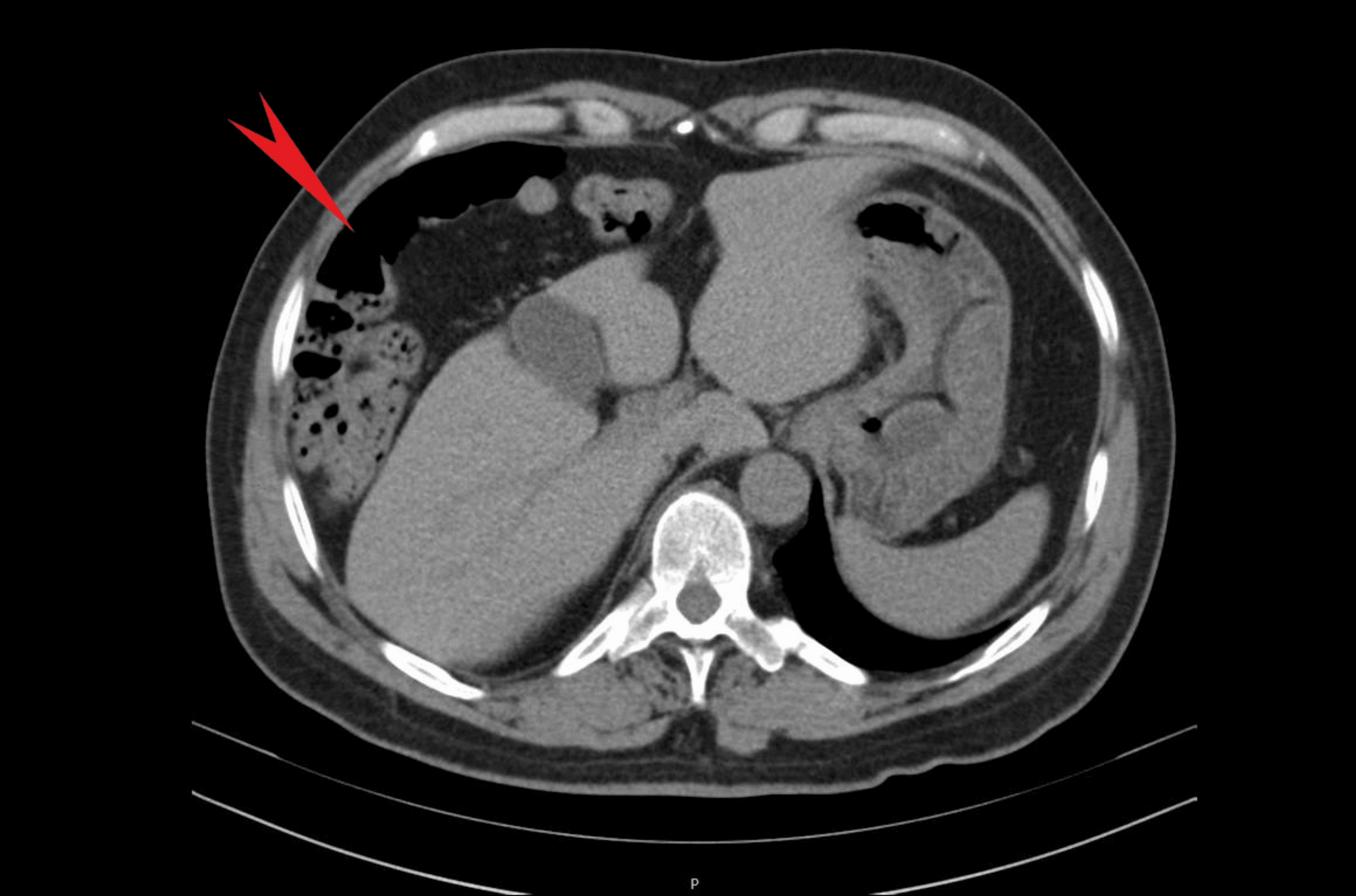
Abdominal CT scan CT abdomen reported Chilaiditi sign with anterior interposition of the colon to the liver (red arrow).

Diagnostic tests of note include a chest X-ray, which showed elevated diaphragmatic domes, blurred diaphragmatic boundaries, and the Chilaiditi sign (Figure 1). A contrast-enhanced thoracic CT scan revealed a small consolidation area in the right costovertebral gutter with an air bronchogram inside and demonstrated the interposition of the colon between the right hemidiaphragm and the upper surface of the liver, suggestive of Chilaiditi syndrome. An abdominal CT scan confirmed the diagnosis of Chilaiditi syndrome (Figure 2), but without complications or signs of intestinal obstruction, although fecalomas were observed in the colon, particularly in the rectum.

The patient was treated with bronchodilator therapy for an asthmatic crisis and with enemas for constipation and the presence of fecalomas. The patient was discharged with a resolution of the condition.

## Discussion

In this case, we report a new case of Chilaiditi’s sign, occasionally observed on abdominal radiological examination. The patient also presented complaints consistent with the presence of Chilaiditi’s syndrome, such as dyspnea, constipation, and abdominal pain.

Its incidence is 0.025% in radiological examinations across all age groups. However, the incidence slightly increases in patients over 60 years old, being more common in men than in women, with a ratio of 4:1 [6,9]. Its incidence can reach 0.3% on plain chest radiographs and 2.4% on chest/abdominal computed tomography scans [10].

In adults, the syndrome is often associated with cirrhosis, ascites, emphysema, hypothyroidism with chronic constipation, hypertension, coronary artery disease, appendicitis, renal ectopia, Cushing’s syndrome [11], schizophrenia, mental retardation, and the use of antipsychotic drugs [12].

A Greek study with 1,440 patients undergoing chest/abdominal computed tomography revealed that increased intra-abdominal fat was the most commonly associated factor with Chilaiditi’s sign [10].

The predisposing factors in this patient included cognitive deficit, obesity, hypertension, and chronic intestinal constipation.

Considering that the patient had mild symptoms, conservative and targeted therapeutic measures were adopted. The case of dyspnea was associated with an exacerbation of the patient’s known asthma. However, there are situations where dyspnea can occur due to this syndrome, and treatment should be tailored to each specific case. Regarding constipation and the presence of fecalomas observed in imaging exams, they were treated with the administration of enemas, which proved effective in relieving abdominal pain and discomfort.

## Conclusions

With this work, we aim to emphasize the importance of recognizing Chilaiditi syndrome. This holds particular importance in distinguishing it from conditions that necessitate urgent surgical intervention, such as pneumothorax, subphrenic abscess, hollow viscus rupture, liver injuries, or retroperitoneal masses. By doing so, unnecessary and invasive interventions can be avoided.

Therefore, we conclude that although Chilaiditi syndrome is a rare condition, it should be considered as a possible differential diagnosis so that the patient receives appropriate treatment with minimal harm.
